# The Therapeutic Potential of Galectin-3 in the Treatment of Intrahepatic Cholangiocarcinoma Patients and Those Compromised With COVID-19

**DOI:** 10.3389/fmolb.2021.666054

**Published:** 2021-05-24

**Authors:** Hao Li, Jianmin Li, Wei Xiao, Yujing Zhang, Yuan Lv, Xing Yu, Jiao Zheng

**Affiliations:** ^1^ Biliary Tract Surgery Laboratory, Department of Hepatobiliary Surgery, Hunan Provincial People's Hospital, the First Affiliated Hospital of Hunan Normal University, Changsha, China; ^2^ Hunan Research Center of Biliary Disease, the First Affiliated Hospital of Hunan Normal University, Changsha, China; ^3^ Department of Pulmonary and Critical Care Medicine, Hunan Provincial People’s Hospital, The First Affiliated Hospital of Hunan Normal University, Changsha, China; ^4^ Department of Medical Administration, Hunan Provincial People’s Hospital, The First Affiliated Hospital of Hunan Normal University, Changsha, China; ^5^ The Key Laboratory of Model Animals and Stem Cell Biology in Hunan Province, School of Medicine, Hunan Normal University, Changsha, China; ^6^ The Key Laboratory of Molecular Epidemiology in Hunan Province, School of Medicine, Hunan Normal University, Changsha, China; ^7^ Department of Drug Clinical Trial, Hunan Provincial People’s Hospital, The First Affiliated Hospital of Hunan Normal University, Changsha, China

**Keywords:** Gal-3, COVID-19, ICC, cancer, therapy

## Abstract

The novel coronavirus pneumonia COVID-19 is characterized by all age susceptibility, which imposes a dramatic threat to the human species all over the world. According to current available data, the cytokine storm appears to be the most life-threatening symptom of severe COVID-19 cases accompanied with lung fibrosis. Galectin-3 (Gal-3), a member of soluble β-galactoside-binding lectin families, has been implicated as a key regulator in various inflammation conditions in addition to its well-documented roles in cancer. The pro-inflammatory activity of Gal-3 in the inflammatory response and lung fibrosis of COVID-19 has been proposed by emerging studies, which suggested that inhibition of Gal-3 may represent a novel treatment approach for COVID-19 patients. Intrahepatic cholangiocarcinoma (ICC) is an aggressive malignancy with poor prognosis. ICC accounts for 10–25% of primary liver cancers with limited therapeutic options, which has higher incidence in Asian countries, particularly in China. Cancer patients, including ICC patients, are highly vulnerable to COVID-19 due to their impaired immune system. It is thus undoubtedly a challenge for our oncology department to establish effective treatment strategies under the influence of the COVID-19 crisis. According to our management procedures in the COVID-19 era, emergency treatment will be applied to ICC patients who are under life-threatening conditions, despite the COVID-19 infection. To the best of our knowledge, the modulatory function of Gal-3 in ICC is still barely explored to date. In order to evaluate the therapeutic potential of Gal-3 for ICC patients or those comprised with COVID-19, we herein report our preliminary investigation into roles of Gal-3 in ICC. Our results exhibited that the expression of Gal-3 was significantly up-regulated in ICC tissues, and a significant correlation was observed between its overexpression and malignant progression of ICC cells. We further discussed the activity and possible molecular mechanisms of Gal-3 in ICC, which may pave the ways for further exploring the possibility of Gal-3 as a potential therapeutic target for treating ICC patients or those with COVID-19-related conditions.

## Introduction

The infection of a novel coronavirus known as SARS-CoV-2 has caused a disease named COVID-19 by The World Health Organization (WHO). Since December 2019, the catastrophic COVID-19 epidemic has caused more than 100 million of respiratory illnesses worldwide that far exceeded the reported cases of Severe Acute Respiratory Syndromes (SARS) and Middle East respiratory syndrome (MERS) (Cevik et al., 2020; Liang et al., 2020; Wu and McGoogan, 2020). Previous studies on other coronaviruses related severe cases revealed that pulmonary inflammation and lung fibrosis are associated with over-expression of pro-inflammatory cytokines, which is similar to the phenomenon of the so-called cytokine storm of COVID-19 patients (Kuiken et al., 2003; Shin et al., 2019; Huang et al., 2020). Therefore, better understanding of the link between inflammation responses and cytokine production may give rise to the development of potential therapeutic targets especially being beneficial in the treatment of COVID-19 patients.

Galectins (Gal), a subfamily of animal lectins, exert central roles in regulation of the immune system in both physiological and pathological processes, which are characterized by an affinity for β-galactoside-containing glycans. Gal-3 is the only member of the chimera-type galectins, contributing to a variety of disease states, such as inflammation (Liu et al., 2012) and cancer (Song et al., 2014). Ever-increasing evidence has demonstrated the presence of upregulated Gal-3 in COVID-19 patients as compared with healthy controls (De Biasi et al., 2020; Tian et al., 2020), which underline the pro-inflammatory activity of Gal-3 in the progression of COVID-19 conditions. Further, a recent single-cell RNAseq analysis on a variety of immune cells from lungs of COVID-19 patients revealed that elevated expression of gal3 in proliferative T cells appears to be correlated with severe condition of COVID-19 patients (Liao et al., 2020). With regard to the molecular mechanism associated with lung fibrosis, several studies reported that gal3 was one of the most up-regulated genes collaborated with fibrotic marker TREM2 and SPP1 in a subset of pro-fibrogenic macrophages (Krasemann et al., 2017; Ramachandran et al., 2019; Zhou et al., 2020). Together with its well-documented roles in cancer, Gal3 has thus been considered as a potential therapeutic target to alleviate the fatal inflammation condition and subsequent lung fibrosis in COVID-19 patients, especially for those compromised with cancers.

Intrahepatic cholangiocarcinoma (ICC) is one of the common malignant hepatic tumors with poor prognosis, high recurrence and rapid invasion and metastasis, which poses a serious threat to human health (Rahnemai-Azar et al., 2017). In recent years, the incidence of ICC has demonstrated a significant increase in many countries, with China accounting for more than half of the global incidence rate (Bridgewater et al., 2014; Rizvi et al., 2018). To our knowledge, the modulatory function of Gal-3 in ICC is still quite limited. According to the COVID-19 management procedures in our hospital, emergency treatment will be applied to ICC patients who are under life-threatening conditions, despite the COVID-19 infection. In view of the lack of a treatment strategy of cancer patients infected with COVID-19 as well as the reasonable rationale of utilizing galectin-3 as a novel therapeutic target, we have investigated the role and possible regulatory mechanisms of Gal-3 in ICC. Our results showed that malignant behavior of ICC cells were significantly inhibited after Gal-3 knockdown, accompanying with decreased expression of MMP9, a key regulator in cancer and inflammation progression. Our study not only for the first time systematically discussed the role and possible molecular mechanisms of Gal-3 in ICC, also provided experimental basis for further exploring the possibility of Gal-3 as a potential therapeutic target for treating ICC patients or those compromised with COVID-19.

## Materials and Methods

### Data Mining

The RNA-Seq data of Gal-3 were obtained and analyzed from UALCAN (http://ualcan.path.uab.edu/analysis.html).

### Reagents and Tissue Specimen

HCCC9810 cells (ATCC); DMEM medium, fetal bovine serum (FBS) and trypsin (Gibco, Grand Island, United States); penicillin-streptomycin solution (HyClone); Gal-3 silent expression and control lentivirus and HitransG P virus infection reagent (Jikai gene, Shanghai, China); RIPA lysate, phosphatase inhibitor mixture, phenylmethyl sulfonyl fluoride, diquinolinecarboxylic acid (bicinchoninic acid, BCA) protein quantitative kit and 5 × sodium dodecyl sulfate (5 × SDS) (Beyotime, Shanghai, China); skim milk powder and TBST (Dingguo Changsheng Biotechnology, Beijing, China); Gal-3, MMP9, proliferating cell nuclear antigen (proliferating cell nuclear antigen, PCNA), Cyclin D1, p21 and glyceraldehyde phosphate dehydrogenase (Cell Signaling Technology, Danvers, MA, United States); HRP-FITC labeled secondary antibody (Wuhan Sanying Biotechnology); PVDF membranes (Millipore, Billerica, MA); Western Blot hypersensitive chemiluminescence reagent (Thermo, MA, United States); ImageJ software (Media Cybernetics, MD, United States); MTT and DMSO (Sigma); Trizol (Invitrogen, MA, United States); PrimeScriptTM RT Regeant Kit and SYBR Premix Ex TaqTM (TAKARA); 5-ethynyl-2-fluoro-deoxyuridine (5-ethynylmurine) cell proliferation detection kit (Solebao Technology, Beijing, China). 24 ICC and paracancerous tissue samples were obtained from ICC patients prior to any preoperative chemo- and radiotherapy, or other medical interventions at the Department of Hepatobiliary Surgery, the First Affiliated Hospital of Hunan Normal University.

### Immunohistochemistry

ICC and paracancerous tissues were fixed with formalin and embedded in paraffin to make 5 μm sections, and the specimens were treated overnight in an oven at 60 C. After dewaxing and alcohol soaking, specimens were rinsed with water, then soaked in 10% hydrogen peroxide and further rinsed with phosphate buffer (PBS). Antigen repair was done at 95 C for 30 min, then rinsed with PBS after cooling to room temperature. The primary antibody of Gal-3 (1:500) was incubated overnight at 4 C, and the second antibody was incubated at room temperature for 30 min. The film was developed using 3,3′-diaminobenzidine (DAB) at room temperature for 15 min and photographed.

### Cell Culture

HCCC9810 cells were cultured at 37 C in DMEM medium containing 10% FBS and 1% penicillin-streptomycin at 37 C in a 5% CO2 incubator. Cells of 10–30 generations were used in the experiment.

### Lentivirus Infection

Lentivirus infection was inoculated on a 6-well plate with a density of 5 × 10^4^ cells per well. The cells were divided into a control group and a Gal-3-shRNA group. The target sequence used to generate shRNAs against the open reading frame of Gal-3 mRNA was 5′- TGC​CTC​GCA​TGC​TGA​TAA​CAA-3′ to silence Gal-3. After 16 h of inoculation, the cells were transfected according to the manufacturer’s instructions (2 ml DMEM medium containing 2 μL lentivirus and 40 μL HitransG P transfection reagent). After inoculation, the cells were screened for about 7–10 days.

### Quantitative Real-Time PCR Assays and Western Blot

The total RNA of the control group and the Gal-3-shRNA group was extracted by Trizol and the concentration was determined. The PCR reaction system was prepared according to the manufacturer’s instructions. Primer sequence: ACTB (forward 5′- CAC​CAC​GCG​TGA​TGG​T-3′; reverse 5′-CTC​AAG​CAT​GAT​CTG​GGA​CAT-3′; Gal-3 (forward 5′-TAT​TCC​GTG​TAT​AGT​CAC​CGG-3′; reverse 5′-TGC​AAG​CTT​GAA​GTG​GTC​AG-3′; Bcl-2 (forward 5′-GCG​GAG​ATT​GGG​ACA​ACA​GCT​GTA-3′; reverse 5′-GAC​GCG​CCT​GAA​CAC​CCA​CA-3′). Western blot was carried out according to the manufacturer’s instructions. Briefly, 2 ×  10^5^ cells were scraped and lysed with RIPA lysis buffer (Beyotime, Wuhan, China). The lysates were then collected at 12,000 g for 15 min at 4 C. And the concentrations of the total proteins were quantified. Protein samples were then separated with 10–15% SDS-PAGE gel and transferred onto the PVDF membranes. Immune complexes were formed by incubation of proteins with primary antibodies (Cell Signaling Technology, Danvers, MA, United States) overnight at 4 C followed by incubation with horseradish peroxidase conjugated second antibody (ABclonal Technology, Wuhan, China) for 1 h at room temperature. Immunoreactive protein bands were detected with ImageJ software.

### MTT, Colony Formation and Edu Assay

MTT, colony formation and Edu assay were performed according to the manufacturer’s instructions. In brief, the absorbance (OD) of MTT was measured colorimetrically at a wavelength of 490 nm for 0, 24, 48, 72, 96, and 120 h, respectively. The cell growth curves were plotted with the number of observation days as the horizontal coordinate and the OD value as the vertical coordinate. The number of colony formation was counted and the morphology was observed under an inverted microscope, and a cell cluster larger than 50 cells was considered as one clonal colony. In Edu assay, the nuclei were incubated for 30 min at room temperature with anti-Edu working solution, washed with PBS and stained with Hoechst 33,342 at a concentration of 1 μmol/L for 15 min, then observed under a fluorescent inverted microscope and photographed.

### Wound Healing and Transwell Assay

Wound healing and Transwell assay were performed according to the manufacturer’s instructions. In brief, the cells used for wound healing assay were observed by microscope and photographed at 0, 24 and 48 h, respectively, and the scratch area was calculated using ImageJ software based on the corresponding scratch area change rate. The transfected cells used in Transwell assay were inoculated into the chambers above the 24-well plate at a density of 5 × 10^4^ cells, and the cells were replaced with 150 µL of serum-free medium after overnight wall attachment. 200 µL of complete medium was added to the lower 24-well plate, and the chambers were removed after 24 h. The cells were fixed with 4% paraformaldehyde at room temperature for 30 min, stained with crystal violet staining solution at room temperature for 15 min followed by rinse of water, then observed under a microscope and photographed.

### Statistical Analysis

Statistical analyses were performed using SPSS 19.0. Each experiment was repeated 3 times, and the experimental results were expressed as mean ± SD. The correlation analysis was examined using Dunnet t test. The *p* < 0.05 was considered statistically significant.

## Results

### Expression of Gal-3 in Intrahepatic Cholangiocarcinoma

**FIGURE 1:**
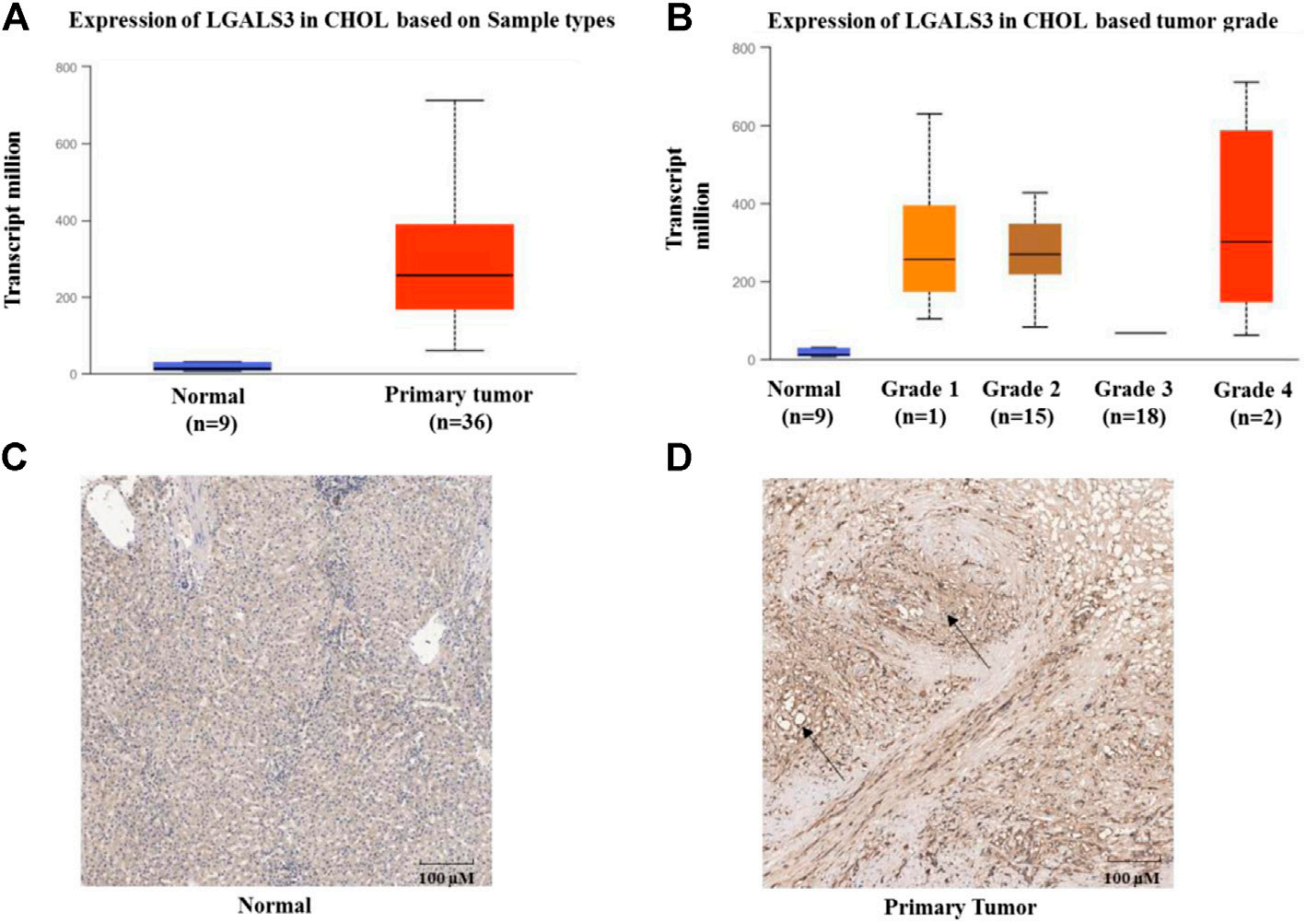
Expression of Gal-3 in ICC **(A)** Expression of Gal-3 based on sample types **(B)** Expression of Gal-3 based on cancer stages **(C)** Expression of Gal-3 in paracancerous tissues **(D)** Expression of Gal-3 in ICC tissues. UALCAN was used to analyze TCGA data of cholangiocarcinoma in Figures 1A,B. *Y*-axis denotes the expression level of gene of interest, while *X*-axis denotes groups of control (Normal, nine samples) and patients (36 samples). Staining of Gal-3 in Figure 1D was highlighted by black arrows.

Based on the TCGA data mining (UALCAN), mRNA levels of Gal-3 between ICC and paracancerous tissues were compared as shown in Figures 1A,B. The mRNA level of Gal-3 was significantly higher in ICC tissues than that of paracancerous tissues (Figure 1A, *P*< 0.001). In addition, Gal-3 mRNA level was positively correlated with ICC progression (Figure 1B; paracancerous tissues compared to ICC Stage 1, *P*< 0.0001 and paracancerous tissues compared to ICC Stage 2, *P*< 0.001). The protein expression levels of Gal-3 in ICC and paracancerous tissues were further examined using immunohistochemistry (IHC) (Figures 1C,D), which demonstrated that Gal-3 was markedly increased in ICC tissues (*P*< 0.05).

### Knockdown of Gal-3 Inhibits the Proliferation Ability of Intrahepatic Cholangiocarcinoma Cells

**FIGURE 2:**
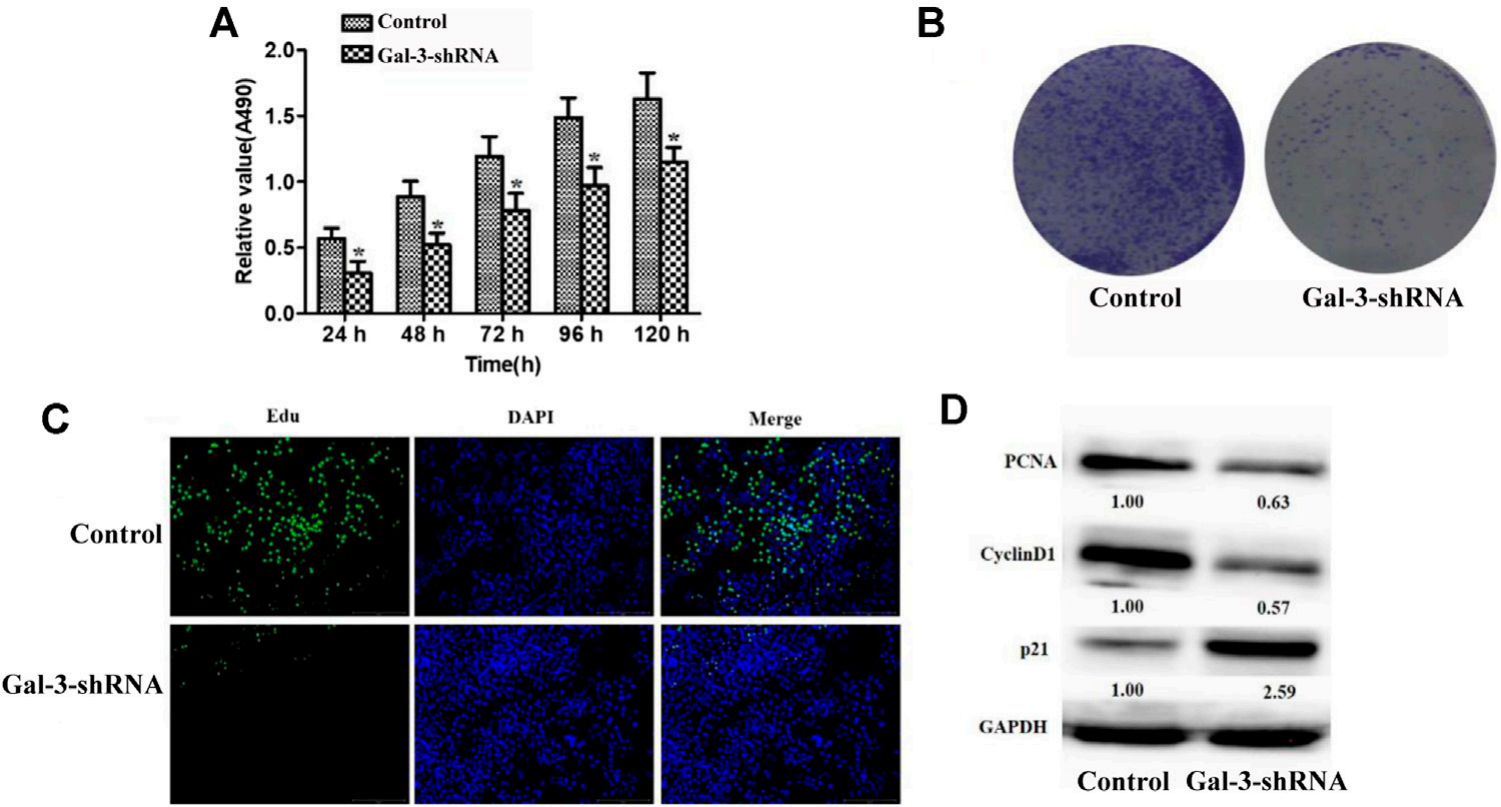
Knockdown of Gal-3 inhibits the proliferation ability of ICC cells **(A)** MTT assay was utilized to assess the proliferation activity of ICC cells **(B)** Decreased number of colonies formed was observed after Gal-3 expression was depleted **(C)** The number of Edu-positive cells was decreased after Gal-3 knockdown **(D)** The protein expression level of PCNA, Cyclin D1, and p21 was examined.

In order to evaluate the effect of Gal-3 upon cell proliferation in ICC cells, we deprived its expression in ICC HCCC9810 cells by shRNAs. Gal-3 was effectively suppressed in ICC HCCC9810 cells compared to the control, which was validated by our qRT-PCR and Western blot results (Supplementary Figure S1). MTT assay was then utilized to assess the proliferation activity of ICC HCCC9810 cells. As seen in Figure 2A, our results indicated depletion of Gal-3 led to impaired proliferation capacity observed at time points between 24 and 120 h. The colony formation assay also showed that the number of colonies formed as well as relevant clonal area was significantly diminished compared to the control group (Figure 2B). In addition, decreased number of Edu-positive cells in the Gal-3-shRNA group was observed (Figure 2C). To further explore the biological role of reduced Gal-3 in proliferation of ICC cells, expression levels of proliferation-related proteins were determined. PCNA and Cyclin D1 were down-regulated after knockdown of Gal-3, contrasting that p21 protein expression was elevated more than two fold (Figure 2D). Together, those results suggested that Gal-3 has an essential role in modulation of proliferation of ICC cells.

### Low Expression of Gal-3 Inhibits Metastasis of Intrahepatic Cholangiocarcinoma Cells

**FIGURE 3:**
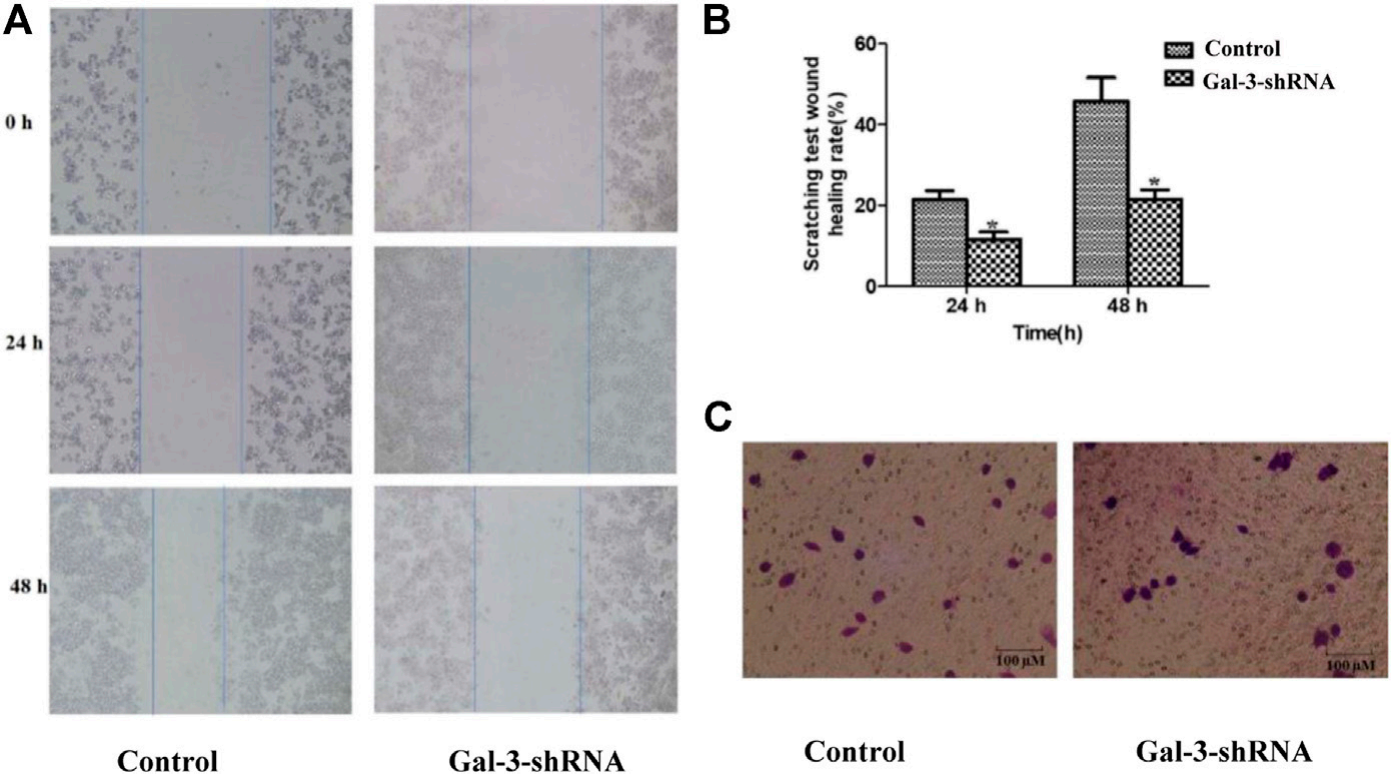
Low expression of Gal-3 inhibits metastasis of ICC cells. The impact of Gal-3 upon the invasion and metastasis ability of ICC cells was examined by wound healing assay **(A, B)** and Transwell assay **(C)**.

Similar to other malignant tumors, metastasis is one of the typical clinical features of ICC. The impact of Gal-3 upon the invasion and metastasis ability of ICC cells was thus examined by performing wound healing assay and Transwell assay (Figure 3). Our result showed that ICC cells in the Gal-3-shRNA group exhibited 9.81 and 24.21% decreased migration ability compared with the control group after 24 and 48 h, respectively, which was well correlated with the result of Transwell assay showing that ICC cells transfected with Gal-3-shRNA exhibited decreased migration ability of crossing the membrane in contrast to the control group.

### Down-Regulation of MMP9 is Mediated by Gal-3 Knockdown

**FIGURE 4:**
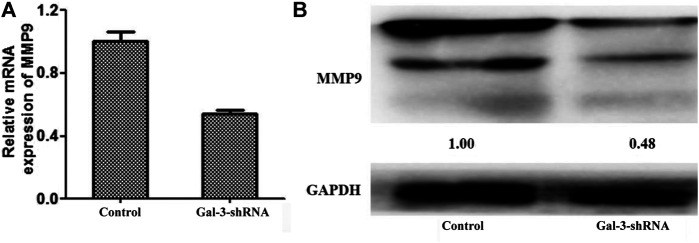
Down-regulation of MMP9 is mediated by Gal-3 knockdown. The mRNA and protein levels of MMP9 were detected by qRT-PCR **(A)** and Western blot **(B)** in ICC cells transfected with Gal-3-shRNA.

The matrix metalloproteinase (MMP) family is one of the key regulators in degradation and remodeling of the extracellular matrix and the basement membrane, which is essential for a variety of malignant tumors to facilitate their metastasis. Previous studies revealed that MMP9 was significantly associated with poor survival and lymph node metastasis in ICC (Shirabe et al., 1999; Sirica et al., 2009) and various inflammation states (Borjini et al., 2019; Jing et al., 2020). The mRNA and protein levels of MMP9 were thus detected by qRT-PCR and Western blot in ICC cells (Figure 4). Compared with the control group, the mRNA and protein levels of MMP9 were significantly diminished in HCCC9810 cells transfected with Gal-3-shRNA, suggesting that MMP9 may act as a downstream target of Gal-3.

## Discussion

Patients with cancers appear to be most vulnerable to the effects of the coronavirus pandemic, of particular when they have other treatment-related complications. It is therefore of upmost importance to establish an effective management approach in the treatment of cancer patients during the COVID-19 era. According to our emergency procedures in the COVID-19 era, urgent treatment will be applied to patients who are under life-threatening conditions, despite the COVID-19 infection. Further, as fever is one of the most common symptoms of cancer patients, an imperative focus during the pandemic is to identify whether the fever is a treatment-related symptom or COVID-19 related. As seen in Supplementary Figure S2 of the consultation record, a 24/7 multidisciplinary joint-telemedicine consultation platform and a multi-channel patient-centered process have been constructed in our hospital to ensure the effective sorting for patients affected by possible travel restrictions. Patients with life-threatening conditions were then arranged for immediate hospital admission. ICC is the second most common malignant hepatic tumor prone to early invasion and metastasis, with an incidence of approximately 10–25% among primary tumors of the liver (Shaib et al., 2004; Wang et al., 2013). In recent years, incidence and mortality rate of ICC have remarkably increased, especially in China, which seriously affects human life and health. Hence the lack of effective treatment approaches of this malignancy imposes an urgent demand to better understand the oncogenesis of ICC in attempt to develop novel therapeutic strategies, which could also benefit ICC patients compromised with COVID-19.

Mammalian Gal-3 is a 35 kDa protein coded by the *LGALS3* gene located on chromosome 14 (Dong et al., 2018). Gal-3, a glycan-recognizing protein mainly found in the cytoplasm and cell membrane, participates in a wide range of cellular signaling processes and regulates biological events such as inflammation, cancer, cell proliferation, cell cycle and apoptosis (Gonnermann et al., 2020; Schroeder et al., 2020; Song et al., 2020). Evidence indicated that Gal-3 was ubiquitously expressed in virtually all immune and inflammatory cell types, such as epithelial cells, dendritic cells, macrophages and neutrophils. Therefore, Gal-3 was regarded as a potent inflammatory protein closely related to both acute and chronic inflammation contributing to the initiation and amplification of the inflammatory response. In the acute inflammatory response, Gal-3 could regulate the chemoattraction of monocytes/macrophages (Subhash et al., 2016), neutrophil clearance (Karlsson et al., 2009), opsonization of apoptotic neutrophils (Wright et al., 2017) and mast cell degranulation (Bambouskova et al., 2016). Chronic inflammation acts as a key regulator in a variety of diseases, including cardiovascular diseases, cancer, diabetes, arthritis, Alzheimer's disease, pulmonary diseases, and viral infections. As aforementioned, Gal3 has been considered as a potential therapeutic target to alleviate the fatal inflammation condition and subsequent lung fibrosis in COVID-19 patients. Therefore, our aim was next to investigate the role of Gal-3 in progression of ICC cells and relevant regulatory mechanisms, which may shed some light on potential applications of Gal-3 in treating ICC. Accumulating evidence indicates that high expression of Gal-3 is associated with the development of various malignancies (Dolgormaa et al., 2020; Li et al., 2020). In our study, the expression data of Gal-3 in ICC were retrieved from the TCGA database, and its correlation with the clinical stages of ICC was investigated. We found that the mRNA level of Gal-3 was markedly elevated in ICC tissues and positively correlated with ICC clinical stages. Further, the IHC experiment indicated that the protein level of Gal-3 was significantly increased in ICC tissues in comparison to the control group, which was consistent with the outcome of our Gal-3 mRNA expression analysis.

Previous studies showed that the proliferation ability of cancer cells was significantly reduced after knockdown of Gal-3 in various cancer types (Lalancette-Hebert et al., 2012; Ajani et al., 2018; Yang et al., 2020). Our data also demonstrated that inhibition of Gal-3 dramatically suppressed the proliferation and differentiation ability of ICC HCCC9810 cells as evidenced by increased cell ploidy time. A significant decrease in the number of Edu-positive cells was also observed after Gal-3 expression was depleted, and a similar trend was shown in our colony formation assay result. After knockdown of Gal-3 in ICC cells, the protein expression level of PCNA and positive cell cycle regulator Cyclin D1 that both are closely related to cell proliferation was diminished, while the expression level of negative cell cycle regulator p21 was elevated. Taken together, our experimental data firmly stated that Gal-3 plays an essential role in modulation of ICC cell proliferation.

Several lines of evidence have indicated that one of the strongest prognostic factors associated with survival outcome of ICC patients was attributed to lymph node metastasis (Giuliante et al., 2016; Zheng et al., 2020). Our results showed that the invasive migration ability of ICC HCCC9810 cells with decreased Gal-3 expression was significantly impaired, which correlates well with previous reports. To further explore roles of Gal-3 in regulating invasive phenotypes of ICC cells, we investigated a potential Gal-3-mediated mechanism of ICC cell migration and invasion. MMP9 plays a crucial role in ICC invasion and metastasis, and its association with Gal-3 in tumor progression has been previously stated (Woulfe et al., 2017; Zile et al., 2019). In our study, the mRNA and protein levels of MMP9 in ICC HCCC9810 cells transfected with Gal-3-shRNA were then examined, which demonstrated that the expression of MMP9 was significantly inhibited. It has to be noted that the specific molecular mechanism of Gal-3 influencing the expression of MMP9 needs to be further explored in subsequent studies.

In conclusion, our study demonstrated that Gal-3 is a crucial regulator in the progression of ICC. Our data also implicated that Gal-3 may exert its function, partly if not all, through the MMP9 signaling pathway, suggesting that Gal-3 may be a potential therapeutic target for ICC. To further examine the expression of downstream genes of Gal-3, including MMP9, is our next aim. Of significance is that the possible direct COVID-19 impact on ICC is yet to be elucidated. In addition to that, further investigation is necessary to evaluate the therapeutic potential of Gal-3 for ICC patients or those comprised with COVID-19 despite well-documented roles of Gal-3 in inflammation and cancer.

## Data Availability

Publicly available datasets were analyzed in this study. This data can be found here: http://ualcan.path.uab.edu/cgi-bin/TCGAExResultNew2.pl?genenam=LGALS3&ctype=CHOL.
