# Supplementary figures and images for: The Therapeutic Potential of Galectin-3 in the Treatment of Intrahepatic Cholangiocarcinoma Patients and Those Compromised With COVID-19
Source: Front Mol Biosci. 2021 May 24;8:666054. doi: 10.3389/fmolb.2021.666054 (PMC8180910; doi:10.3389/fmolb.2021.666054)

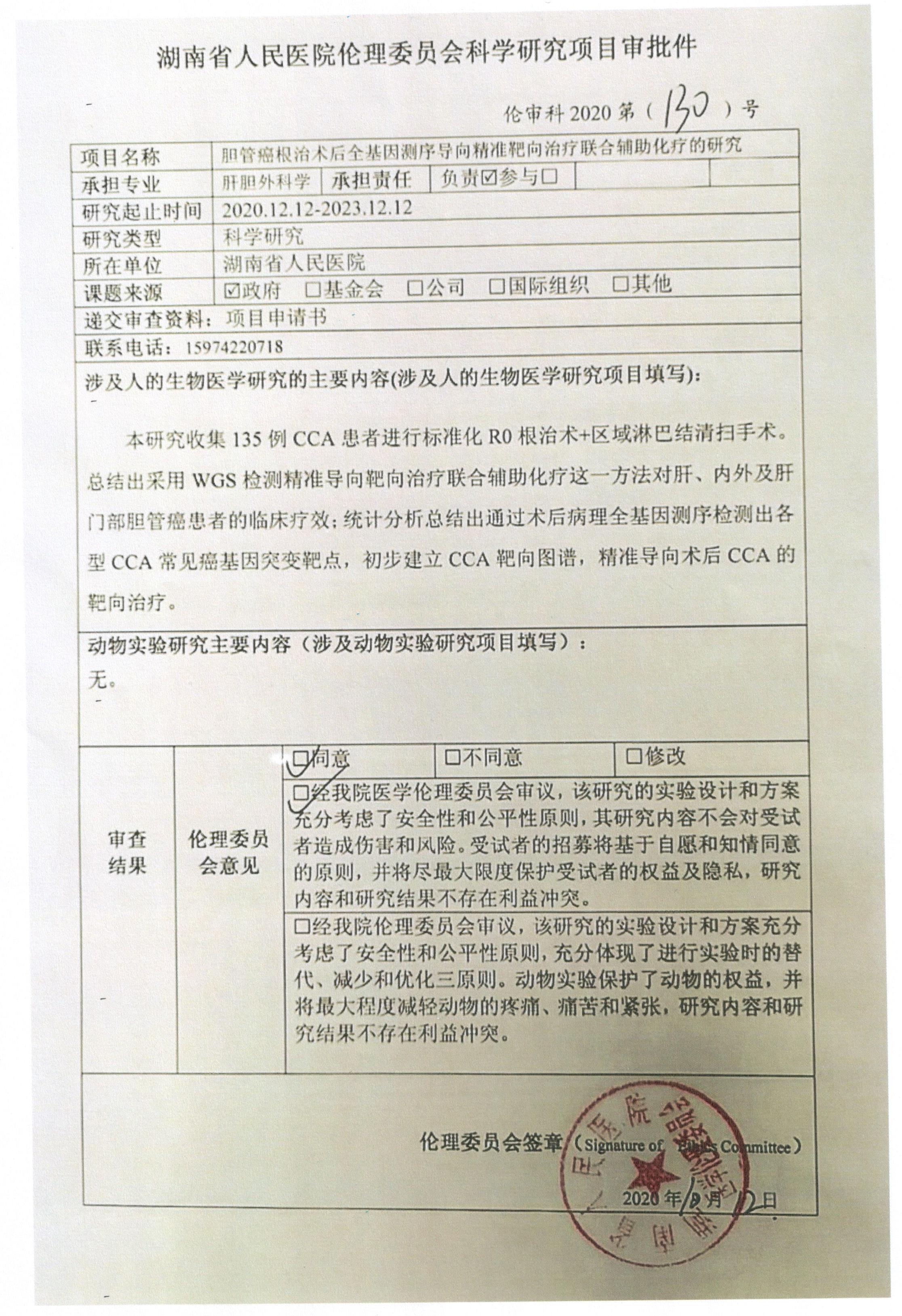

Supplement: Supplementary file 1 [file Image1.JPEG]
