# Supplementary material for: The Therapeutic Potential of Galectin-3 in the Treatment of Intrahepatic Cholangiocarcinoma Patients and Those Compromised With COVID-19
Source: Front Mol Biosci. 2021 May 24;8:666054. doi: 10.3389/fmolb.2021.666054 (PMC8180910; doi:10.3389/fmolb.2021.666054)
Supplement: Supplementary file 2 [file DataSheet1.PDF]

Supplementary Material

Supplementary Figures

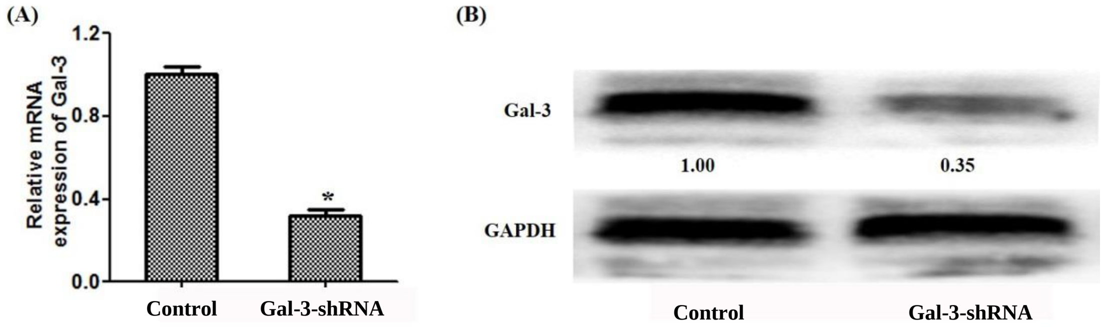

Supplementary Figure 1. Validation of Gal-3 knockdown

(A) Knockdown of Gal-3 significantly suppressed the mRNA level of the Gal-3-shRNA group compared to the control group; (B) Western Blot assay also showed that the protein expression of Gal-3 in the Gal-3-shRNA group was significantly lower than that in the control group.

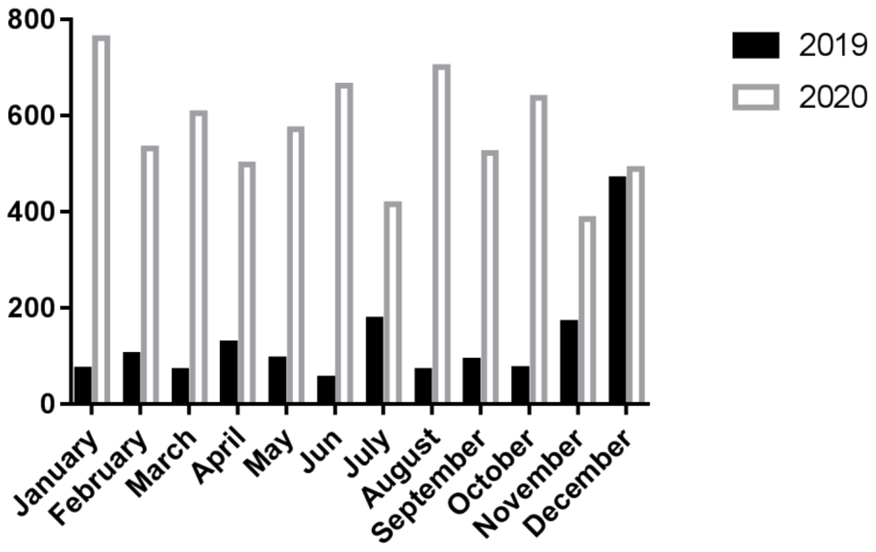

Supplementary Figure 2. Monthly urgent consultations with reported fever

Demonstration of monthly urgent consultations with reported fever during 2019 and 2020.
